# Use of a Web-Based Physical Activity Record System to Analyze Behavior in a Large Population: Cross-Sectional Study

**DOI:** 10.2196/jmir.3923

**Published:** 2015-03-19

**Authors:** Hideyuki Namba, Yosuke Yamada, Mika Ishida, Hideto Takase, Misaka Kimura

**Affiliations:** ^1^Department of Health and NutritionWayo Women’s UniversityIchikawa, ChibaJapan; ^2^National Institute of Health and NutritionTokyoJapan; ^3^Kao CorporationTokyoJapan; ^4^Faculty of Health and Medical SciencesKyoto Gakuen UniversityKyotoJapan

**Keywords:** motor activity, behavior, Internet, computer systems, metabolic equivalent, Japan

## Abstract

**Background:**

The use of Web-based physical activity systems has been proposed as an easy method for collecting physical activity data. We have developed a system that has exhibited high accuracy as assessed by the doubly labeled water method.

**Objective:**

The purpose of this study was to collect behavioral data from a large population using our Web-based physical activity record system and assess the physical activity of the population based on these data. In this paper, we address the difference in physical activity for each urban scale.

**Methods:**

In total, 2046 participants (aged 30-59 years; 1105 men and 941 women) participated in the study. They were asked to complete data entry before bedtime using their personal computer on 1 weekday and 1 weekend day. Their residential information was categorized as urban, urban-rural, or rural. Participant responses expressed the intensity of each activity at 15-minute increments and were recorded on a Web server. Residential areas were compared and multiple regression analysis was performed.

**Results:**

Most participants had a metabolic equivalent (MET) ranging from 1.4 to 1.8, and the mean MET was 1.60 (SD 0.28). The median value of moderate-to-vigorous physical activity (MVPA, ≥3 MET) was 7.92 MET-hours/day. A 1-way ANCOVA showed that total physical activity differed depending on the type of residential area (F_2,2027_=5.19, *P*=.006). The urban areas (n=950) had the lowest MET-hours/day (mean 37.8, SD, 6.0), followed by urban-rural areas (n=432; mean 38.6, SD 6.5; *P*=.04), and rural areas (n=664; mean 38.8, SD 7.4; *P*=.002). Two-way ANCOVA showed a significant interaction between sex and area of residence on the urban scale (F_2,2036_=4.53, *P*=.01). Men in urban areas had the lowest MET-hours/day (MVPA, ≥3 MET) at mean 7.9 (SD 8.7); men in rural areas had a MET-hours/day (MVPA, ≥3 MET) of mean 10.8 (SD 12.1, *P*=.002). No significant difference was noted in women among the 3 residential areas. Multiple regression analysis showed that physical activity consisting of standing while working was the highest contributor to MVPA, regardless of sex.

**Conclusions:**

We were able to compile a detailed comparison of physical activity because our Web-based physical activity record system allowed for the simultaneous evaluation of physical activity from 2046 Japanese people. We found that rural residents had greater total physical activity than urban residents and that working and transportation behaviors differed depending on region type. Multiple regression analysis showed that the behaviors affected MVPA. People are less physically active while working, and sports and active transportation might be effective ways of increasing physical activity levels.

## Introduction

Despite known hazards, physical inactivity continues to be a major risk for the development of chronic diseases [1]. It is associated with an increased incidence of obesity, diabetes, cardiovascular disease, osteoporosis, and cancer [2-4]. Therefore, instruments that allow for accurate measurement of physical activity in the general population are needed. Further, accurately determining what factors affect physical activity is crucial. Measurement of physical activity via the Internet may help to compensate for the weaknesses of previous measurement methods.

In epidemiological studies, questionnaires are the most frequently used instruments with which physical activity and energy expenditure are estimated [5]. However, the validity of traditional self-reported questionnaires is lower than that of the gold standard measurement technique: the doubly labeled water (DLW) method [6]. Accelerometers are readily available and have adequate memory for long-term data collection. Although the accelerometer is an excellent tool for physical activity assessment, it is not accurate for cycling, swimming, or activities involving only the upper limbs [5]. Furthermore, its cost precludes it from being suitable for epidemiological studies that require physical activity or energy expenditure measurements in large populations. The DLW method is one of the most accurate and valid systems for evaluating total energy expenditure under free-living conditions [7]. However, it is too costly for epidemiological studies. Therefore, it is necessary to develop new and inexpensive methods for evaluating the physical activity of large populations.

Three recent studies compared physical activity measurements between Web-based systems and the DLW method and found that the validity of the Web-based systems was equivalent to that of traditional questionnaires [8-10]. The simplified recording of physical activity every 15 minutes showed high validity compared with the DLW method with respect to energy expenditure [11,12]. We also developed a Web-based physical activity measurement system and compared its accuracy with that of the DLW method [10]. In our system, participants chose their behavior from a menu on the left of the screen and then filled in a table on the right side based on recollection of their behaviors for each 15-minute interval throughout a 24-hour period.

The use of Web-based measurement systems has been suggested as an easier method for collecting self-reported physical activity data than traditional questionnaires. These measurement systems are low cost, practical, and data can be collected quickly (input time of less than 10 minutes). Web-based measurement systems can measure daily physical activity in many people simultaneously because they are compatible with multiple Web browsers and computer operating systems that can be used to complete the assessments via the Internet. The successful and systematic collection of demographic and lifestyle data are central to any epidemiological study [13]. Studies using questionnaires have shown that neighborhoods and social environments are associated with physical activity [14-16]. In the United States, reports showed that rural residents had lower levels of physical activity than urban residents, and this was suggested to be an important contributor to their relatively poorer health [17,18]. However, another recent study that used accelerometer-measured physical activity indicated that total physical activity was higher in rural residents compared with urban residents, but that the reverse was true when considering high-intensity activity [19]. Although epidemiological studies that use accelerometers to collect physical activity data from large populations of people could be done by a national organization, they could also be done using the Internet at a much lower cost. Because comparing urban and rural physical activity has yielded conflicting results, examining subcategories of behavior might be informative.

The purpose of this study was to collect behavioral records from a large population using our Web-based physical activity system and compare the physical activity of people living in residential environments that differed in how urban/rural they were.

## Methods

### Recruitment

A total of 2566 people aged 30-59 years, including 1438 men (56.04%) and 1128 women (43.96%), participated in this study. A request to participate in this survey was made to 9134 of approximately 4,450,000 people who had registered with an Internet research company between July 12 and August 8, 2012. When registering, people were required to provide personal information, such as their residential area, age, and sex. We requested that the Internet company send surveys to 9134 of their registered customers based on our criteria for residence, age, and sex. Of the 9134 people who received a cross-sectional survey, 2566 responded (28.09% cooperation rate). The activity records included 1 weekday and 1 weekend day.

All participants were categorized by age, sex, and residential area. Residential areas were divided into urban (cities around Tokyo), urban-rural (cities with a population >300,000), or rural (municipalities with a population <50,000). The Internet research company provided a URL address in an email and requested that each participant fill out a survey. Each participant received the equivalent of approximately US $3 for completing the survey. The study was conducted with approval by the ethics committees of Fukuoka University in Fukuoka prefecture and Wayo Women’s University in Chiba, Japan. All participants read an explanation of the purpose and content of the study by email and indicated their understanding before providing written consent (via email) to participate.

### Web-Based Physical Activity Records

**Figure 1:**
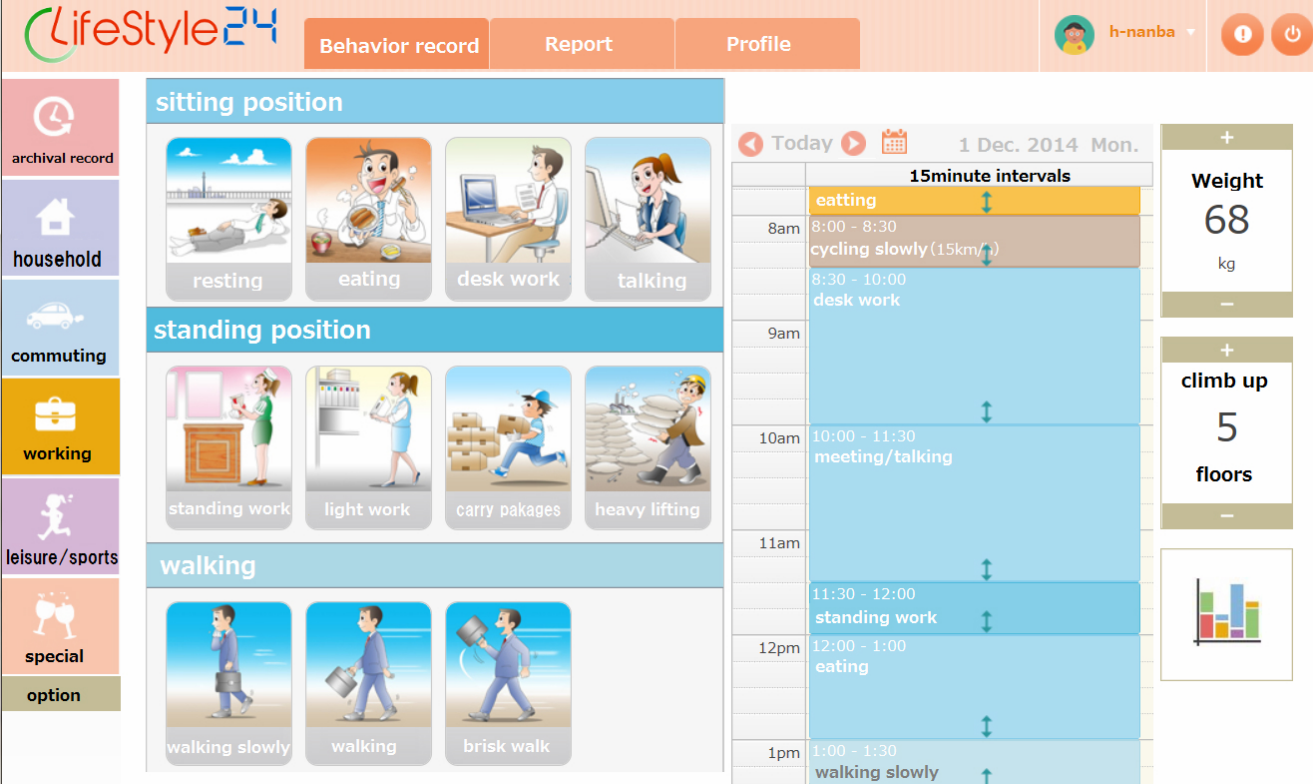
Screenshot of the Web-based physical activity record system.

Figure 1 shows the screen of the Web-based physical activity records system [20]. Participants were requested to respond before bedtime on 2 days (1 weekday and 1 weekend day) using their personal computer. The Web-based record used the diary method [11] to collect the recalled activities over the last 24 hours. Using this method, the participants recorded preset activities performed in each of 4 different categories (household, transportation, work-related, and leisure/sports) for each 15-minute interval of a 24-hour day for cross tabulation. Participants could choose from 91 activities among the 4 categories. We used the intensity of each activity as measured in metabolic equivalent (MET) [21] to quantify participant responses. Thus, the data used for analysis were two 24-hour records of MET values, each with 96 values (every 15 minutes).

**Figure 2:**
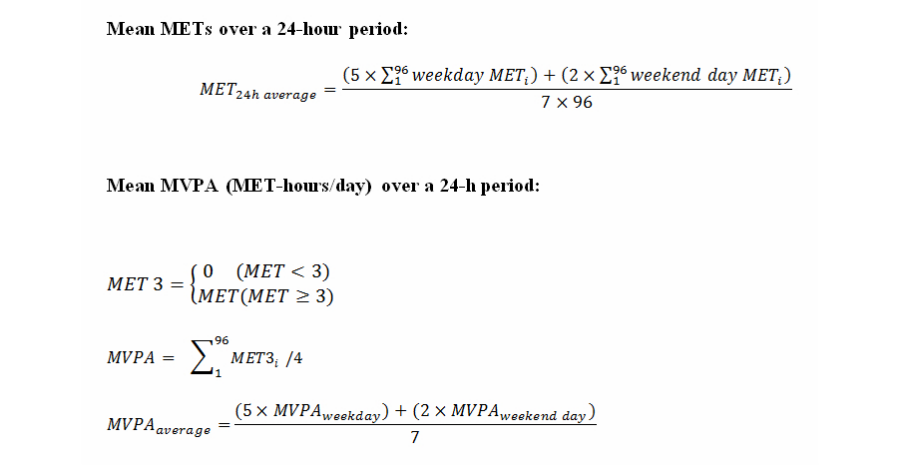
Equations for calculating mean metabolic equivalents (METS) and moderate-to-vigorous physical activity (MVPA) over a 24-hour period.

The mean MET over a 24-hour period was calculated as (weekday × 5 + weekend day × 2) / 7 using equations listed in Figure 2. We also calculated the mean moderate-to-vigorous physical activity (MVPA, MET-hours/day) over a 24-hour period using a similar formula (Figure 2). In this case, we summed MET ≥3, as defined by the EPAR2006 guidelines for MVPA in Japan [22].

### Statistical Analysis

All statistical analyses were performed using SPSS for Windows 20.0 (IBM Corp, Armonk, NY, USA). All data are shown as mean and standard deviation (SD). Comparison of residential area type (a general characteristic) was performed by 1-way ANOVA and chi-square test. Comparison of the total physical activity, MVPA, and the 4 categories of physical activity in each region was performed by 1-way ANOVA and 1-way ANCOVA, and multiple comparisons were subsequently carried out by Tukey honestly significant difference test. A significant difference was defined as *P*<.05. We also compared time spent commuting and working depending on residential area using a 1-way ANCOVA. Multiple regression analysis was performed to investigate the impact of the behaviors on total physical activity (MET-hours) and on the 24-hour MVPA (MET-hours) for the total sample, men, and women. The standardized coefficients (βs) were calculated.

## Results

Table 1 displays the characteristics of the study population. As intended, we recruited a heterogeneous group (3 residential areas, both sexes, and a wide age range), and this is demonstrated in the table. Of the 2566 participants, 520 (20.26%) were excluded from analysis because they did not provide complete datasets (one 24-hour weekday and one 24-hour weekend day). The regional breakdown for the remaining 2046 participants was 950 in urban areas (46.43%), 432 in urban-rural areas (21.11%), and 664 in rural areas (32.45%).

**Table 1:** Characteristics of the study population (N=2046).

Characteristics		Urban (n=950)	Urban-Rural (n=432)	Rural (n=664)	*P*
Age (years), mean (SD)		43.9 (7.9)	43.4 (7.2)	43.6 (7.4)	.47
Weight (kg), mean (SD)		61.3 (12.7)	62.0 (12.6)	62.3 (13)	.27
BMI (kg/m^2^), mean (SD)		22.3 (3.5)	22.7 (3.5)	22.3 (3.7)	.13
**Gender, n (%)**					
	Men	511 (53.8)	220 (50.9)	374 (56.3)	.21
	Women	439 (46.2)	212 (49.1)	290 (43.7)	
**Age range (years), n (%)**					
	30-39	331 (34.8)	156 (36.1)	221 (33.3)	.003
	40-49	326 (34.3)	174 (40.3)	280 (42.2)	
	50-59	293 (30.8)	102 (23.6)	163 (24.5)	
**Custom of periodical exercise, n (%)**					
	No	621 (65.4)	272 (63.0)	443 (66.7)	.09
	>30 minutes twice a week	329 (34.6)	160 (37.0)	221 (33.3)	
**Occupation, n (%)**					
	Employee	522 (54.9)	238 (55.1)	342 (51.5)	.07
	Self-employed	59 (6.2)	28 (6.5)	62 (9.3)	
	Full-time housewife	142 (14.9)	56 (13.0)	78 (11.7)	
	Housewife (includes part-time job)	114 (12.0)	47 (10.9)	82 (12.3)	
	Student	4 (0.4)	1 (0.2)	0 (0.0)	
	Unemployed	50 (5.3)	28 (6.5)	37 (5.6)	
	Other	59 (6.2)	34 (7.9)	63 (9.5)	
**Household income (million yen), n (%)**					
	<3	123 (12.9)	86 (19.9)	121 (18.2)	<.001
	3-5	204 (21.5)	129 (29.9)	198 (29.8)	
	5-7	245 (25.8)	103 (23.8)	175 (26.4)	
	7-10	217 (22.8)	68 (15.7)	103 (15.5)	
	>10	161 (16.9)	46 (10.6)	67 (10.1)	

There were no significant differences among the 3 regions with respect to mean age (*P*=.47), weight (*P*=.27), or BMI (*P*=.13). Detailed characteristics of the study population were also investigated. These included gender, age, exercise habits, occupation, and household income in each respective region. We found no differences among the 3 regions with respect to gender, exercise habits, or occupation. However, some significant differences were found with respect to age. Among the age group between 50-59 years, 30.8% (293/950) were from urban areas, 23.6% (102/432) from urban-rural areas, and 24.5% (163/664) from rural areas (*P*=.003). Additionally, household income was significantly higher in urban areas than in other regions (*P*<.001).

**Figure 3:**
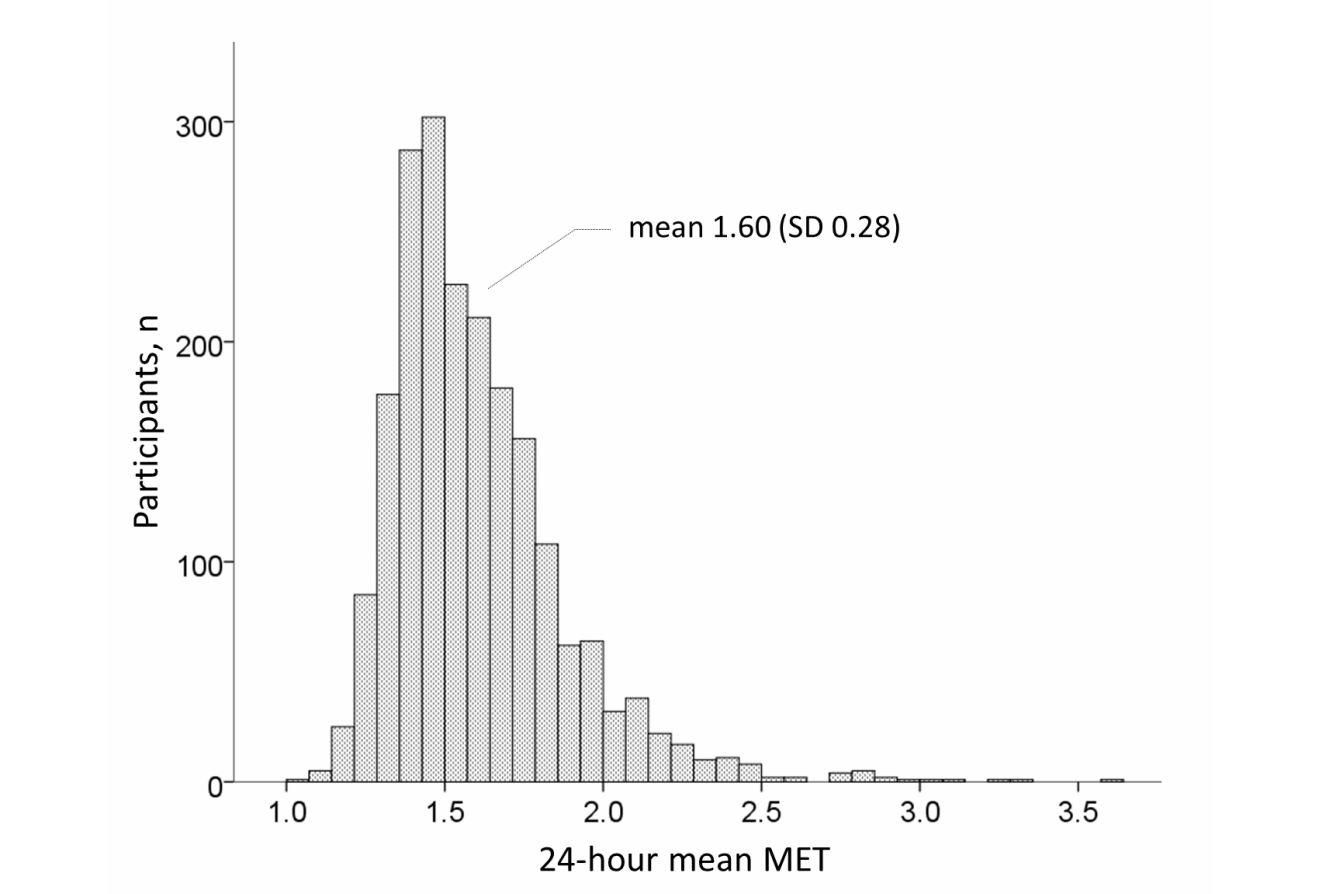
Distribution of 24-hour mean metabolic equivalents (MET) for 2046 participants.

**Figure 4:**
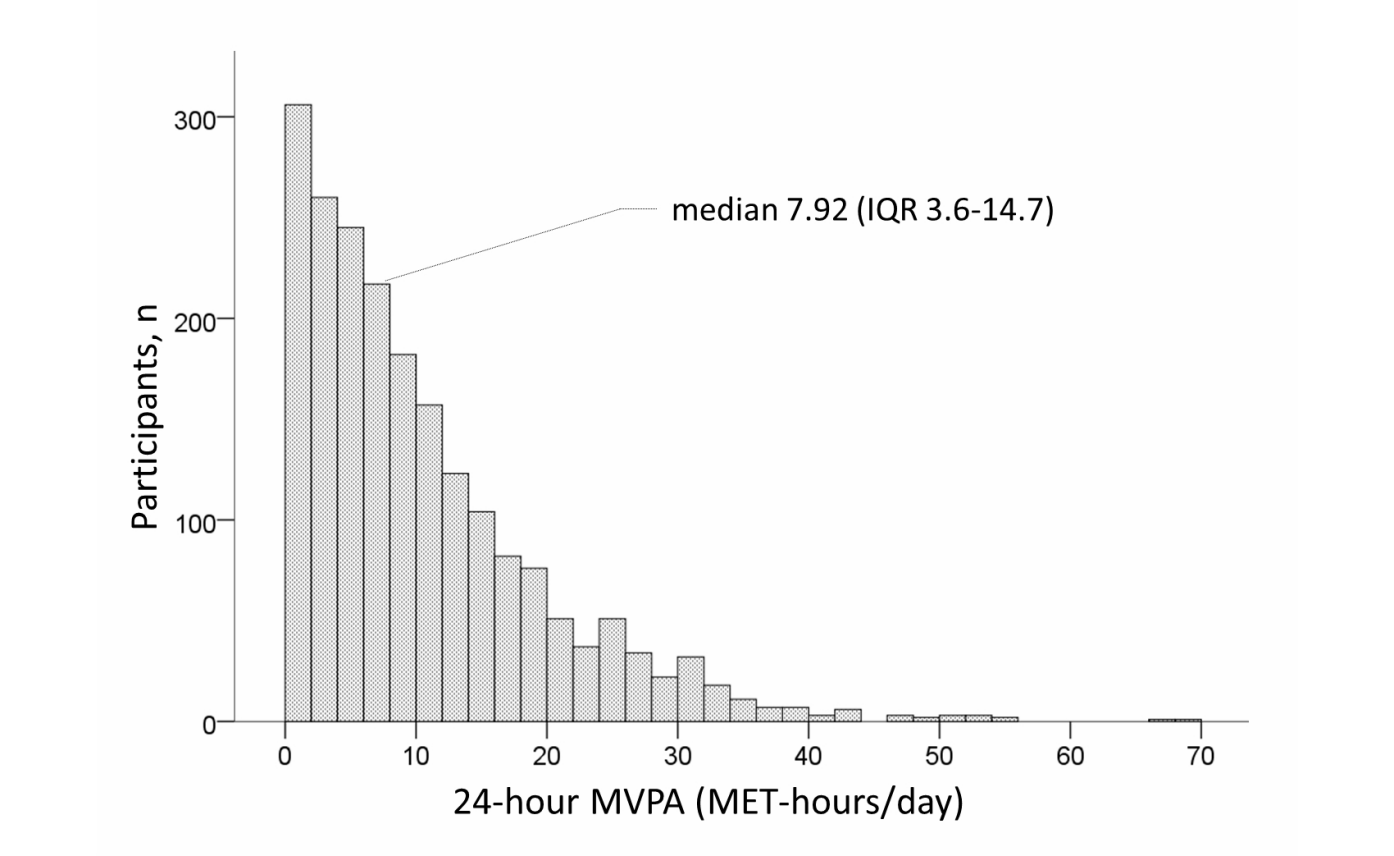
Distribution of 24-hour moderate-to-vigorous physical activity (MVPA) for 2046 participants.

Figure 3 shows the distribution of the 24-hour mean MET values among all participants. Most participants had a mean MET between 1.4 and 1.8, and the mean 24-hour MET was 1.60 (SD 0.28). Figure 4 shows the MVPA distribution among all participants. Most participants had an MVPA between 0 and 10 MET-hours; the median 24-hour MVPA value was 7.92 MET-hours (IQR 3.6-14.7).

Table 2 compares total physical activity, MVPA, the 4 categories of physical activity, and detailed categorical activity across region type. One-way ANOVA revealed significant differences in total physical activity (24-hour mean MET) depending on area of residence (*F*_2,2043_=7.26, *P*<.001), and a 1-way ANCOVA showed that when adjusted for age, gender, occupation, household income, and marriage, the total physical activity (MET-hours/day) differed across region type (*F*_2,2027_=5.19, *P*=.006). Multiple comparison analysis showed that urban areas had the lowest 24-hour mean MET (mean 1.57, SD 0.25), followed by urban-rural areas (mean 1.62, SD 0.27; *P*=.02), and rural areas (mean 1.63, SD 0.31; *P*<.001). No significant differences were found in MVPA across regions.

A 1-way ANOVA of the 4 categories of activity showed that transportation (*F*_2,2043_=32.87, *P*<.001) and working (*F*_2,2043_=17.75, *P*<.001) significantly differed depending on region type. When we broke the transportation category down into subcategories, a 1-way ANCOVA revealed that the amount of time spent walking to work (*F*_2,2028_=115.8, *P*<.001), on public transit (*F*_2,2028_=179.4, *P*<.001), and sitting in a car (*F*_2,2028_=89.51, *P*<.001) significantly differed across region type. Multiple comparison analysis showed that urban areas had the longest walking time, followed by urban-rural and rural regions (urban: mean 24.6, SD 25.1 min; urban-rural: mean 7.8, SD 24.9 min; *P*<.001; rural: mean 7.4, SD 25.0 min; *P*<.001). Time spent sitting in cars was lowest in urban areas (mean 21.9, SD 52.0 min; *P*<.001), followed by urban-rural areas (mean 51.5, SD 51.9 min) and rural areas (mean 52.8, SD 51.9 min). There were no significant differences in time spent sitting at work among the 3 region categories. Time spent standing at work was longest in rural areas (mean 89.7, SD 152.4 min; *P*<.002) vs urban-rural, followed by urban-rural areas (mean 67.0, SD 125.5 min; *P*<.001) vs urban, and urban areas (mean 44.7, SD 106.2 min; *P*<.001) vs rural.

Two-way ANCOVA adjusted for age, occupation, household income, and marriage showed a significant interaction between sex and area of residence on the urban scale (*F*_2,2036_=4.53, *P*=.01), and a significant main effect was observed for sex (*F*_1,2036_=20.98, *P*<.001). Men in urban areas had the lowest MET-hours/day (mean 7.9, SD 8.7); men in rural areas had a mean MET-hours/day of 10.8 (SD 12.1, *P*=.002). No significant difference was noted in women among the 3 residential areas.

**Figure 5:**
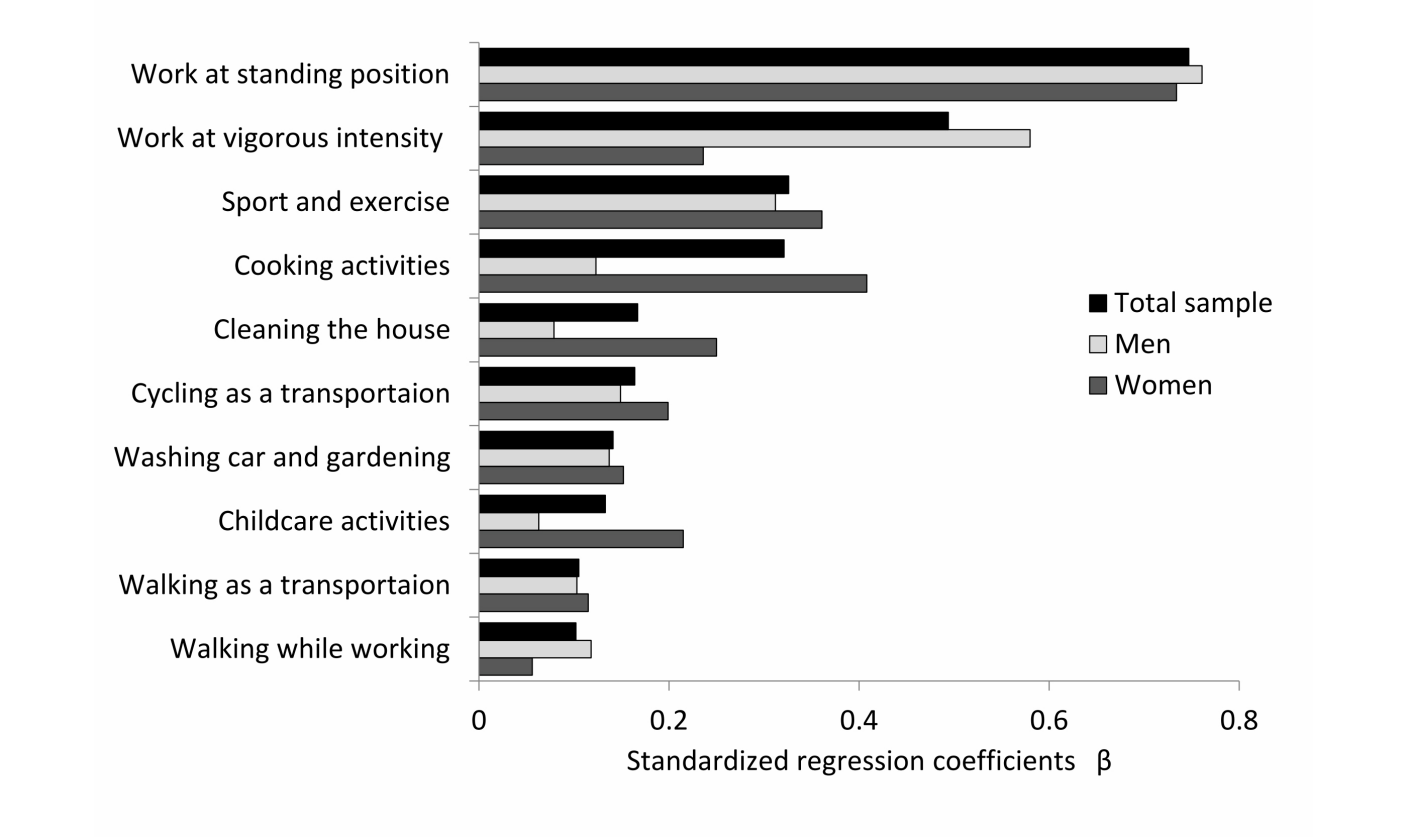
Multiple regression analysis for moderate-to-vigorous physical activity in men and women.

Figure 5 shows the results of the multiple regression analysis for MVPA (MET-hours/day, ≥3.0 MET) against the subcategories of activities, and plots the resulting standardized coefficients (βs). We analyzed the total sample (*R* ^*2*^=.95, *P*<.001, standard error of the estimate [SEE]=2.08, n=2046), just the men (*R* ^*2*^=.96, *P*<.001, SEE=2.24, n=1105), and just the women (*R* ^*2*^=.94, *P*<.001, SEE=1.86, n=941).

The independent variable here is physical activity (MET-hours/day) and includes varying intensities. In the total sample, the highest contribution was made by working while standing, followed in order by vigorous work, sport and exercise, cooking, cleaning the house, cycling as transportation, washing the car and gardening, childcare, time spent walking on the way to work, and time spent walking during work. Physical activities that were weighted differently between men and women were vigorous working, cooking, cleaning the house, and childcare.

**Table 2:** Comparison of physical activity in each region type.

Types of physical activity			Urban, mean (SD) (n=950)	Urban-Rural, mean (SD) (n=432)	Rural, mean (SD) (n=664)	*F* (*df*)	*P*
**Total physical activity**							
	24-Hour mean MET		1.57 (0.25)	1.62 (0.27)	1.63 (0.31)	7.26 (2,2043)	<.001
	24-Hour MET-hours/day		37.9 (6.0)	38.8 (6.5)	39.1 (7.5)	7.26 (2,2043)	<.001
	24-Hour MET-hours/day^a^		37.8 (6.0)	38.6 (6.5)	38.8 (7.4)	5.19 (2,2027)	.006
**Moderate-to-vigorous physical activity**							
	24-Hour MET-hours/day ≥3.0 MET		9.8 (8.5)	10.7 (9.4)	11.3 (10.6)	5.58 (2,2043)	.04
	24-Hour MET-hours/day^a^ ≥3.0 MET		10.0 (9.3)	10.4 (9.3)	11.2 (9.3)	3.23 (2,2038)	.04
**Categories of physical activity**							
	**Household physical activity**						
		MET-hours/day	5.6 (6.5)	6.5 (7.3)	5.9 (6.4)	2.73 (2,2043)	.07
		Time (min)	118.0 (135.6)	134.0 (150.0)	121.6 (132.0)	2.02 (2,2043)	.13
	**Transportation physical activity**						
		MET-hours/day	4.1 (3.2)	3.1 (3.1)	3.0 (2.7)	32.87 (2,2043)	<.001
		Time (min)	99.7 (78.9)	71.1 (67.8)	73.8 (69.4)	34.16 (2,2043)	<.001
	**Working physical activity**						
		MET-hours/day	8.0 (7.4)	9.5 (9.0)	10.6 (10.4)	17.75 (2,2043)	<.001
		Time (min)	253.0 (202.0)	269.1 (202.7)	277.8 (204.7)	3.06 (2,2043)	.047
	**Leisure/Sports activity**						
		MET-hours/day	9.8 (5.5)	9.4 (4.6)	9.3 (4.9)	2.67 (2,2043)	.07
		Time (min)	394.6 (187.4)	393.6 (190.4)	389.0 (192.8)	0.18 (2,2043)	.84
**Detail of transportation**							
	Walking time while commuting^a^ (min)		24.6 (25.1)	7.8 (24.9)	7.4 (25.0)	115.8 (2,2028)	<.001
	Public transit while commuting^a^ (min)		45.6 (42.4)	7.6 (42.3)	11.9 (42.2)	179.4 (2,2028)	<.001
	Sitting in a car while commuting^a^ (min)		21.9 (52.0)	51.5 (51.9)	52.8 (51.9)	89.51 (2,2028)	<.001
**Detail of working**							
	Sitting time of working^a^ (min)		203.3 (196.9)	213.2 (196.3)	192.7 (185.2)	1.89 (2,2028)	.15
	Standing time of working^a^ (min)		44.7 (106.2)	67.0 (125.5)	89.7 (152.4)	17.77 (2,2028)	<.001

^a^ Adjusted for age, gender, occupation, household income, and marriage.

## Discussion

In this study, we succeeded in simultaneously collecting data via the Internet from 2046 people using the Web-based physical activity system that we developed. The data were used to perform a behavior analysis and comparison of physical activity by residential area type. The percentage of people who fully cooperated with the request to participate in data collection was only 22.4%. Therefore, although large-scale collection of physical activity data is possible using Internet research, challenges remain.

The accuracy of the simplified physical activity record has been confirmed by the DLW method [11,12] and good agreement was observed using the Web-based physical activity measurement system [10]. Although recording behavior using paper requires many procedures, using a Web-based system allows participants to complete the record in a relatively short time and responses from many participants can be collected and managed by the data server simultaneously. Figure 3 shows that the mean 24-hour MET value is similar to the mean value obtained from Japanese participants using the DLW method [23]. Figure 3 indicates that the median 24-hour mean MVPA (MET-hours/day) was 7.92, which we consider to be higher than the mean 25.0 (SD 14.7) MET-hours/week (ie, approximately 3.5 MET-hours/day) reported using an accelerometer in 1837 Japanese participants [24]. Therefore, the MVPA value using the present system may have been overestimated. For example, 10-minute activities would be counted as 15 minutes because our minimum time interval was 15 minutes. Furthermore, participants may have overestimated their actual behavior through inaccurate recall. However, the system described here allows assessment of swimming, cycling, and climbing activities, which cannot be measured using an accelerometer. The risk of overestimating physical activity using the recall method has been previously described [6], but further studies of diary methods are needed to clarify the efficacy of assessment using accelerometers and global positioning systems.

Comparing the activity data with how urban/rural the residential area was showed that people in urban areas had lower MET-hours values than those in rural and urban-rural areas. Associations of neighborhood walkability attributes with commuting by walking have been confirmed in Australia [14]. With respect to environment, physical activity has been associated with transportation, population density, recreational activities, and connectivity [25]. In this study, although the time spent walking to work among participants in urban areas was a mean 24 (SD 25.3) minutes, it was only a mean 7.4 (SD 25.3) minutes in rural areas. Time spent commuting by car or public transportation has been shown to differ significantly between urban and rural residents. That people in urban areas had higher physical activity related to transportation than did those living in rural areas is consistent with data from the National Health and Nutrition Examination Survey (NHANES) [26].

The duration of time spent standing while working was shorter in urban areas than in urban-rural and rural areas. This can be explained by the fact that urban areas exhibited lower MET values and potentially less time standing. The duration of time spent sitting while working among men was longer in urban than in rural areas. This can be explained by the fact that men in urban areas exhibited lower MVPA values and potentially longer sedentary times. Therefore, there is a possibility that work behavior, when such behavior accounts for most of the day, may affect the physical activity of the day. However, no difference in the MVPA according to city size was observed in women. Occupational activity significantly contributes to overall physical activity in British adults [27]. Researchers in the United Kingdom have shown that deindustrialization is related to a decrease in physical activity in a region [28]. Therefore, when work behavior accounts for most activity within a day, it should have a high impact on the physical activity for that day.

Multiple regression analysis was performed to determine which behaviors affect the MVPA. In the total sample, standing while working was the category that most strongly affected MVPA. Behaviors related to working (standing while working, vigorous working, and walking while working) accounted for 3 of the top 10 behaviors that affected MVPA. Household and childcare activities (childcare, cleaning, and cooking) accounted for another 3, leisure and sport activities (sports/exercise, washing car and gardening) accounted for 2, and commuting (walking and bicycling) accounted for the remaining 2 (Figure 5). Therefore, to increase physical activity, activity while working must be intentionally performed, and doing sports, exercising, commuting, or doing household activities in daily life is important. The idea that work behavior strongly influences physical activity is a perspective consistent with previous research [27-29]. Characteristic physical activities for men included working vigorously, and those for women were cooking, cleaning, and childcare. These activities might be difficult components to change in Japan because they depend on gender cultural roles. Therefore, recommendations regarding sports activities and changes in commuting behavior (ie, switch from driving to cycling or walking) might be useful for preventing lifestyle-related diseases and for improving physical fitness. However, because a recent study demonstrated that health risks are associated with sitting for a long period despite an adequately high MVPA [30], reducing time spent sitting might be an additional point to consider if one wants to increase the 24-hour mean MET.

Some limitations of the present study should be noted. In Japan, more than 95% of people aged 30-49 years and 91.4% of people aged 50-59 years have access to the Internet, whereas only 81.7% of people (all ages) have access to a personal computer [31]. Because the system described here can only be used on a personal computer, the potential use of smartphones should be explored in further studies. The low cooperation rate in this Internet survey might have led to self-selection bias in which those who chose to cooperate were typically either more or less active than those who did not. Combining the Internet version with conventional mailing may be an effective way to improve the cooperation rate.

In conclusion, the present Web-based physical activity records system allowed for the simultaneous evaluation of physical activity in 2046 Japanese individuals. The system also ensured that behavioral data were collected for each 15-minute interval within a 24-hour period. Prior Web-based activity record systems have been developed to select the behavior record of character information [10]; we have developed a method by behavior record illustrations on the Web screen in the new system. New Web and smartphone systems that use illustrations may allow for the spread of such systems to a large number of users. We compared the physical activity of residents in urban vs rural regions, and examined 4 categories of physical activity. We found that working and transportation behavior differed depending on region type. Multiple regression analysis showed that physical activity while working was the factor that most affected MVPA, whereas men and women differed in how much vigorous working, cooking, cleaning, and childcare affected their MVPAs.

Future research should investigate the development of systems for measuring behavioral change based on behavior records. The characteristics of the present Web-based physical activity records system are that it can evaluate details of individual lifestyles over a 24-hour period through the recall of activity in 15-minute intervals. It is important to note that behavior can be changed to improve individual lifestyles.

## Abbreviations

DLW: doubly labeled water
MET: metabolic equivalent
MVPA: moderate-to-vigorous physical activity
SEE: standard error of the estimate
